# Delayed Diagnosis of an Eating Disorder in a Male Patient With Superior Mesenteric Artery Syndrome: Results From a Case Study

**DOI:** 10.3389/fpsyt.2019.00731

**Published:** 2019-10-15

**Authors:** María Recio-Barbero, Sara Fuertes-Soriano, Janire Cabezas-Garduño, Mayte López-Atanes, Alvar Peña-Rotella, Margarita Sáenz-Herrero

**Affiliations:** ^1^Department of Psychiatry, Biocruces Bizkaia Health Research Institute, Cruces University Hospital, Barakaldo, Spain; ^2^Department of Neurosciences, School of Medicine and Nursing, University of the Basque Country (UPV/EHU), Leioa, Spain; ^3^Department of Psychiatry, Cruces University Hospital, Osakidetza-Basque Health Service, Barakaldo, Spain

**Keywords:** eating disorders, superior mesenteric artery syndrome, Wilkie’s syndrome, anorexia nervosa, gender bias

## Abstract

**Background:** Eating disorders (EDs) are serious and life-threatening mental diseases characterized by abnormal or altered eating habits. The prevalence is variable, being influenced by diverse sociocultural factors. Historically, the prevalence of EDs has been higher in women (90%), although the incidence of these disorders in men appears to be increasing. In daily medical practice, when considering the presentation of other medical complications associated to the development of an ED, few is known about its real prevalence in men. Among them, some severe gastrointestinal complications that are rarely presented, such as the superior mesenteric artery syndrome (SMAS), can produce life-threatening results. Despite that, very few cases of men presenting this pathology are reported in literature.

**Case Presentation:** A 38-year-old man without a history of psychiatric disease was admitted to the emergency department with nausea, abdominal pain, and severe malnutrition (body mass index 15.7 kg/m^2^). He was diagnosed with SMAS and was studied by multiple specialists on suspicion of a probable organic origin of his thinning. The suspected diagnosis of ED was rejected for months by some professionals, as well as by the patient and his family, until it was finally diagnosed with unspecified feeding and eating disorder (USFED).

**Conclusion:** This case represents an example of diagnostic challenge where a delayed diagnosis of an ED in a male patient was made probably due to gender bias in clinical research and practice. In the literature, numerous reports were described in women diagnosed with SMAS with a previous diagnosis of an ED; however, few cases were found in men. In this clinical case, the patient suffered a significant diagnostic delay, probably due to the lack of diagnostic suspicion given by the differences in the prevalence and clinical presentation of EDs in women and men.

## Background

Eating disorders (EDs) are serious and life-threatening mental diseases characterized by abnormal or altered eating habits. The prevalence is variable, being influenced by diverse sociocultural factors. Historically, ED has been considered as a “female” disorder, with a higher prevalence presentation in women, with a sex ratio ranging from 3:1 to 18:1 (1). Despite that, some recent data appear to show an increase in the incidence of EDs in men (2).

When considering the presentation of any psychiatric disorders, and specifically in the case of EDs, sex and gender have been pointed out to have a causative influence in the differential patterns of the disease presentation (3, 4). Some authors have highlighted the scarcity of scientific literature regarding the study of EDs in men, exposing as an example that only around 1% of research in anorexia nervosa (AN) has been conducted in males (5). As a consequence, men presenting EDs are frequently underresearched, underdiagnosed, and undertreated (6).

ED clinical symptomatology varies, and diagnosis is frequently based on a wide range presentation of symptoms. Females often present higher scores in body dissatisfaction, bulimia, and thinness behaviors compared to men, whereas men present lower weight and shape concerns (4). Some studies have reported that males report less severe symptoms than females (3, 4).

Most frequently described symptoms in patients with a diagnosis of AN, bulimia nervosa (BN), or binge eating disorder include a wide range of organic complications, including prolonged QTc and arrhythmias, abnormalities of endocrine function, dyspepsia, or the development of gastric diseases (7, 8). Gastrointestinal symptoms, including nausea, vomiting, postprandial fullness, abdominal and gastric distension, epigastric pain, or bloating, among others, are commonly described in ED patients, with rates of presentation ranging from 48% to 96% (9). As a consequence, the presentation of some severe gastrointestinal disorders frequently overlaps with ED symptoms, hampering differential diagnosis and requiring further analysis. Comorbid medical conditions such as dermatological, metabolic, and gastrointestinal disorders may appear among ED patients, so the evaluation and intervention of these patients from a multidisciplinary perspective of the healthcare staff are therefore essential (10).

Furthermore, when considering the presentation of other medical complications associated to the development of an ED, few is known about its real prevalence by gender. Among them, some severe gastrointestinal complications, such as the superior mesenteric artery syndrome (SMAS), can produce life-threatening results, but very few cases are reported in literature (11).

SMAS, also known in the literature as Wilkie’s syndrome, is a rare vascular disorder characterized by an entrapment of the third part of the duodenum due to a reduced angle (< 22°) between the aorta and the superior mesenteric artery (12). The most common cause of SMAS is related to a significant rapid and severe weight loss resulting in considerable decrease of mesenteric fat as a result of diverse medical conditions, surgical procedures, or psychological disorders. The epidemiology of this syndrome is unknown, being more frequently diagnosed in women with an average incidence ranging from 0.2% to 0.78% (13). The most frequent clinical presentation consists of recurrent postprandial pain, nausea, vomiting, bloating abdominal discomfort, or pain and tenderness (9). As clinical signs are not specific, diagnosis is based on radiological techniques.

The case we are about to report illustrates the potential life-threatening consequences of a delayed diagnosis of an ED as a result of gender bias in research and clinical practice.

## Case Presentation

We report a case of a 38-year-old man who first attended the emergency room in August 2017 presenting with blood stained vomit and diffuse epigastric pain.

**Figure 1:**
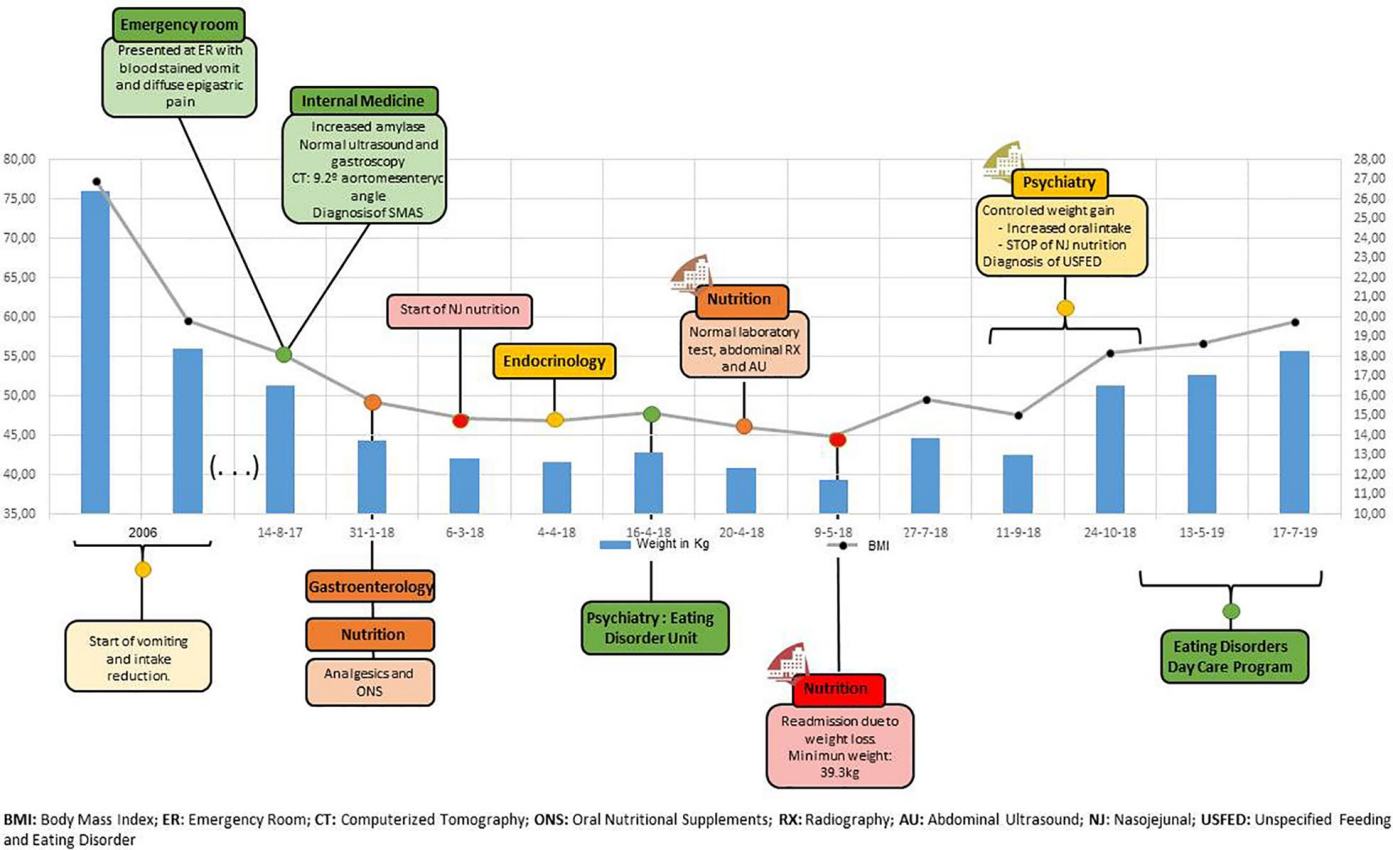
Clinical case timeline.

He had no relevant medical history but described a loss of 20 kg in 2006, for which he had not sought medical attention. At that time, his approximate weight was 76 kg, although no records were found. He started vomiting after dinner and eventually stopped eating. Those symptoms were related by the patient to the stressful situation he was living in his workplace, with problems with his superior and some fellows that eventually led to the patient quitting his job. Ever since he has been unemployed and living in the family home, the weight and frequency of vomiting remained stable until shortly before he attended the emergency department. In **Figure 1**, a visual representation of weight and body mass index (BMI) through the most relevant events is displayed in a timeline.

**Figure 2:**
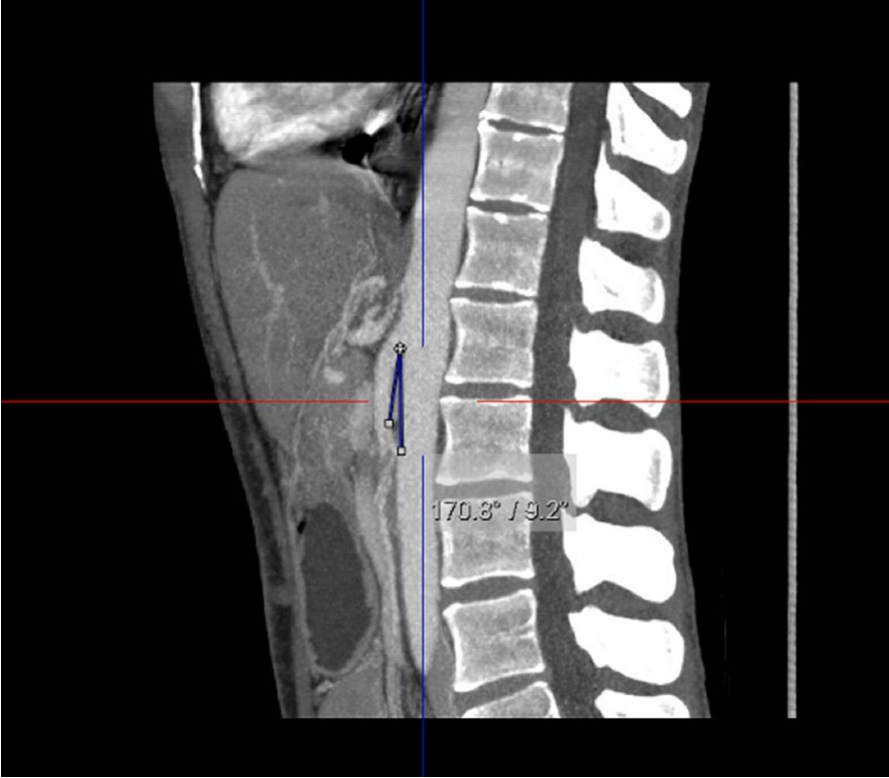
Computerized Tomography of aortomesenteric angle (9.2°)

After ruling out life-threatening conditions, he was referred to internal medicine for additional tests [height (H): 167.5 cm, weight (W): 51.3 kg, BMI: 18.3 kg/m^2^]. Blood analysis revealed an increased serum amylase concentration (137 U/L). The abdominal ultrasound and the gastroscopy showed no pathological findings. The computed tomography (CT) scan, however, described a 9.2° aortomesenteric angle (**Figure 2**), and a diagnosis of SMAS was made based upon these findings.

Surgical treatment was suggested, but as it was not clear whether the SMAS was the cause of the symptom, it was advocated for a conservative course. The patient was referred to gastroenterology and nutrition departments for follow-up, and symptomatic treatment was established with oral nutritional supplements and analgesia. In March, as he appeared to be unable to gain weight, it was decided to start nasojejunal (NJ) nutrition.

A month later, he was first referred to endocrinology in order to rule out an ED, and subsequently to the Eating Disorders Unit, where on the first psychiatric assessment, cluster A personality traits were described. Unfortunately, despite NJ nutrition, due to continued weight loss and vomiting persistence, he had to be shortly admitted at the hospital, delaying psychiatric diagnosis and treatment. After 5 days, where several follow-up tests were conducted (with normal laboratory test, abdominal radiography, and ultrasound results), he was discharged home with joint evaluation and ongoing management by psychiatry, endocrinology, gastroenterology, and nutrition.

During the first psychiatric follow-up visits, a complete anamnesis of the most relevant clinical and biographical data was recorded. He is the youngest of three brothers and sisters. Their parents got divorced in his early childhood, and he has always lived with his mother. His father died 7 years ago of lung cancer, but they were never close. He was described by his family as having a “peculiar” personality; he is sensitive, and he does not take criticism well. The patient described himself as a lonely and shy person. He has had difficulties since early adulthood developing intimate relationships. He is reluctant to confide in others or reveal personal information even to his family. Thus, reference to dysfunctional schizoid personality traits was made through his biography, and supported by the results of the Symptom Checklist 90-R test, a diagnosis of a schizoid personality disorder was made. The eating behavior–related tests, however, were not supportive of an ED diagnosis (Eating Attitudes Test score, 15; Body Shape Questionary score, 39).

Even though he firmly maintained that he was following medical recommendations and denied vomits, his weight continued steadily to diminish to a nadir of 39.3 kg. Consequently, he was soon after readmitted at the Nutrition Department. During this hospital stay in May, psychiatric consultation was requested, and under the presumption of an ED, the patient was transferred to the Psychiatric Ward for closer monitoring. Both the patient and his family denied an eating behavior problem and insisted on an organic origin of the weight loss, denying any psychological aspects to his illness; therefore, he demanded and was granted a voluntary discharge within hours.

From May to August 2018, his weight ranged from 46 to 42 kg. Once his body weight started once again to decrease, he acknowledged self-induced vomiting and agreed to a new hospital admission at the Psychiatric Ward. At this point, his weight was 42.5 kg, and his BMI was 15.24 kg/m^2^. He was hospitalized from September 10 to October 24, being finally diagnosed with an unspecified feeding and eating disorder (USFED) (*Diagnostic and Statistical Manual of Mental Disorders* 307.50) as body image disturbances were never reported by the patient.

In the course of that time, he gained 8.8 kg. During the hospital admission, olanzapine up to 10 mg/d was introduced to regulate sleep patterns and decrease anxiety levels, as the patient referred vomiting to control anxiety. At the same time, anxiolytic (lorazepam 3 mg/day) and antidepressant (fluoxetine 20 mg/day) treatment was added with good tolerance. As he started to increase oral intake, enteral nutrition was gradually decreased until it was finally suspended on October 3. The therapeutic plan during admission consisted of psychoeducation and progressive exposure to food, in order to modify the restrictive eating behavior that has led to chronic malnutrition. At discharge (W: 35.4 kg, BMI: 18.3 kg/m^2^), he started attending daily an Eating Disorders Day Care Program, where he continues until the present day. Throughout the follow-up visits, a psychotherapeutic intervention was carried, both at personal and group levels, jointly by the nutritionist, psychologist, and psychiatrist. His weight remains stable at 55 to 57 kg, and restrictive and purgative behaviors have decreased progressively during these months.

## Discussion

This article presents a case of a male patient with SMAS in which the diagnosis of an ED was delayed. SMAS symptoms are unspecific and can be presented as food intolerance with nausea and vomiting, weight loss, early satiety, abdominal distension, and epigastric pain.

In this case, the patient was assessed by different medical specialists for 8 months before an ED was considered. During this time, multiple tests were performed, including an abdominal CT, which showed a reduction of the aortomesenteric angle. As clinical features were unspecific, they were attributed to SMAS. Due to the important weight loss, enteral nutrition was started. Nevertheless, symptoms got worse, leading to an alarming BMI. At this point, an ED was included in the differential diagnosis, but both the patient and his family were reluctant to accept this option at first. Months later, his condition continued deteriorating until they accepted a psychiatric hospitalization.

One of the most important advancements in the understanding of EDs is the recognition of the severe and prolonged dietary restriction that can lead to serious physical and psychological complications. Many of the symptoms once thought to be primary features of AN are actually symptoms of starvation. In 1944, 36 male volunteers participated in a 13-month study of starvation. The study, known as The Minnesota Starvation Experiment (14), has been cited as one of the most important studies on the mental, physical, and social effects of food restriction. The rapid deterioration, the alarming changes in men’s behavior, and the long-lasting effects of starvation are similar to those seen in EDs. Despite the stereotype that EDs occur only in women, men also exhibit these disorders, and subclinical ED behaviors are as common as in women.

Men with EDs are less likely to seek psychiatric help, and when they do, they are less frequently diagnosed with an ED (15). In fact, there is a lack of awareness among clinicians, and men with EDs are frequently underdiagnosed, undertreated, and thus underresearched.

AN is a condition that predominantly involves females, but it does not exclusively affect women. Epidemiological studies carried out in ED samples estimate a 1:10 prevalence ratio for males, while some data suggest a prevalence up to 25% of the cases, the gap being even closer when considering other specific ED behaviors (16, 17).

Frequently, men diagnosed with an ED may face stigmatization from their peers due to gender stereotypes. Qualitative research has shown that men do present greater difficulty in disclosing ED problems, as being a male does not fit the societal perceptions of the disorder (18). Furthermore, there is a lack of knowledge about how men experience assigned “female” ED perceptions due to the cultural construction of EDs as uniquely or frequently being considered a female disorder. The widespread perception that only women suffer from ED delays the search for help and support.

On the other hand, men could also be underdiagnosed since clinicians fail to recognize gender disparities in presentation. Among EDs, men are less likely than women to present a diagnosis of AN or BN, being the majority of them diagnosed with “other specified eating disorder” or USFED. A possible explanation for this could be that international guidelines fail to take into account gender perspective. It has been described that men with EDs are usually more concerned about achieving a muscled body rather than the female “thin ideal” (18). Nowadays, AN and BN seem to be indisputably associated with women, to the point that all prevention campaigns are developed with a clear bias linked to female gender. Tests do not evaluate behaviors surrounding masculinity, and this fact could be a case of failure to capture ED pathology in men (4).

Despite the efforts made to integrate the gender perspective in biomedical research, there is a lack of knowledge of how gender can mediate and influence the development and course of mental disorders. More specifically, most studies carried out in psychiatry did not take into account how gender-related variables can influence mental health. As a direct consequence, most scientific research in this area has been found to be sex and gender biased (19).

Women and men face social norms and have to attend to gender stereotypes in order to fit in an increasingly more demanding society (20). Therefore, from a biopsychosocial medical paradigm, it seems reasonable that the sociocultural conditions in which men and women live directly affect health outcomes.

Individuals are subject not only to ED gender stereotypes, but also on how they differ in their ability to perceive the inner disease. Authors who reviewed and explored how gender stereotypes impact perceptions of AN confirm that gender affects the perception of AN, in that they do not conform to expectations within the normative model (21).

As stated above, the nonspecificity of the SMAS symptoms may difficult and delay the diagnosis of an ED. The diagnosis of SMAS should be cautiously made as it may simply be a consequence rather than the cause of weight loss, masking a possible ED. Nevertheless, in this particular case, we believe that the diagnosis delay was mainly determined by the influence of gender bias in clinical practice. As clinicians, we should be aware of gender biases that are present in our daily practice and of men being at greater risk to be diagnosed or to experience a diagnostic delay when suffering an ED, which can lead to long-term and life-threatening consequences. It is also important to consider a multidisciplinary overview of the clinical case, considering that an effective communication among physicians is key to achieve optimal outcomes. Future gender-based research is needed to determine the presentation and course of EDs in men in order to an earlier recognition and treatment of these disorders.

## Data Availability Statement

All data collected for this study is included in the manuscript/Supplementary Files.

## Ethics Statement

This study was performed in accordance with the provisions of the Declaration of Helsinki 2008. Written informed consent was obtained from the patient for the publication of this case report. Approval from OSI Ezkerraldea-Enkarterri-Cruces Ethics Committee was obtained for the publication of this research.

## Author Contributions

MS-H and SF-S observed the patient and collected data. ML-A and AP-R collected additional information about the clinical case, including relevant information about the pathobiography of the patient. MR-B and JC-G analyzed the data and performed a bibliography revision of the literature. MR-B, SF-S, JC-G, ML-A, AP-R, and MS-H wrote the paper. All authors approved the final work.

## Conflict of Interest

The authors declare that the research was conducted in the absence of any commercial or financial relationships that could be construed as a potential conflict of interest.
